# Comparison of clinical scores in their ability to detect hypoxemic severe OSA patients

**DOI:** 10.1371/journal.pone.0196270

**Published:** 2018-05-07

**Authors:** Eric Deflandre, Nicolas Piette, Vincent Bonhomme, Stephanie Degey, Laurent Cambron, Robert Poirrier, Jean-Francois Brichant, Jean Joris

**Affiliations:** 1 Department of Anesthesia, Clinique Saint-Luc of Bouge, Namur, Belgium; 2 Department of Anesthesia, University of Liege, Liege, Belgium; 3 Cabinet Medical ASTES, Jambes, Namur, Belgium; 4 University Department of Anesthesia and ICM, CHR Citadelle, Liege, Belgium; 5 Sleep Laboratory Centre (CETES), University of Liege, Liege, Belgium; Wayne State University, UNITED STATES

## Abstract

**Background:**

Severe obstructive sleep apnea (sOSA) and preoperative hypoxemia are risk factors of postoperative complications. Patients exhibiting the combination of both factors are probably at higher perioperative risk. Four scores (STOP-Bang, P-SAP, OSA50, and DES-OSA) are currently used to detect OSA patients preoperatively. This study compared their ability to specifically detect hypoxemic sOSA patients.

**Methods:**

One hundred and fifty-nine patients scheduled for an overnight polysomnography (PSG) were prospectively enrolled. The ability of the four scores to predict the occurrence of hypoxemic episodes in sOSA patients was compared using sensitivity (Se), specificity (Sp), Youden Index, Cohen kappa coefficient, and the area under ROC curve (AUROC) analyses.

**Results:**

OSA50 elicited the highest Se [95% CI] at detecting hypoxemic sOSA patients (1 [0.89–1]) and was significantly more sensitive than STOP-Bang in that respect. DES-OSA was significantly more specific (0.58 [0.49–0.66]) than the three other scores. The Youden Index of DES-OSA (1.45 [1.33–1.58]) was significantly higher than those of STOP-Bang, P-SAP, and OSA50. The AUROC of DES-OSA (0.8 [0.71–0.89]) was significantly the largest. The highest Kappa value was obtained for DES-OSA (0.33 [0.21–0.45]) and was significantly higher than those of STOP-Bang, and OSA50.

**Conclusions:**

In our population, DES-OSA appears to be more effective than the three other scores to specifically detect hypoxemic sOSA patients. However prospective studies are needed to confirm these findings in a perioperative setting.

**Clinical trial registration:**

ClinicalTrials.gov: NCT02050685.

## Introduction

Obstructive Sleep Apnea (OSA) is a risk factor of perioperative complications. This risk increases with the severity of OSA [1], and becomes significant only in severe OSA patients (sOSA) [2]. OSA is defined as severe when the Apnea Hypopnea Index (AHI), i.e. the number of apnea and hypopnea per hour of sleep, equals or exceeds 30 events per hour [3]. AHI is classically obtained during an overnight polysomnography (PSG).

Hypoxic events in OSA patients may be responsible for several pathophysiological modifications [4–12]. Hypoxemia potentially contributes to the development of a coronary artery disease in OSA patients [13], and hence increases the risk of postoperative cardiovascular complications [4–8, 14, 15]. We recently reported that nocturnal episodes of hypoxemia occurred in some but not all sOSA patients [16–18]. Patients combining these two risk factors (severe OSA and hypoxemia) have probably a higher postoperative risk. This hypothesis is based however on data reported outside the postoperative setting. Accordingly, this combination increases the risk of kidney dysfunction and of diabetic retinal complications in humans [19, 20], as well as cardiovascular remodeling in a mouse model of sleep apnea [21]. Consequently, preoperative screening to detect patients combining sOSA and nocturnal hypoxemia may be important for the anesthesiologists [16].

**Fig 1:**
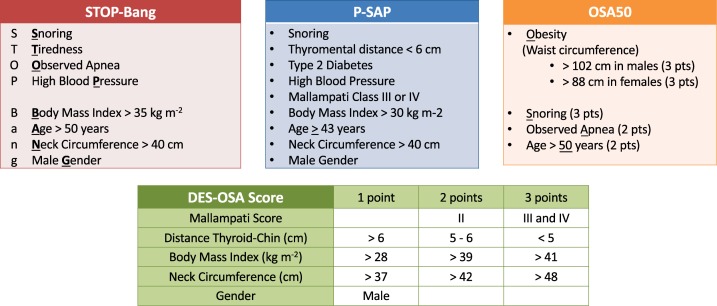
Parameters used in four predictive scores of OSA: STOP-Bang, P-SAP, OSA50, and DES-OSA.

Several predictive scores have been proposed and validated to detect OSA patients preoperatively. The four scores most often used in anesthesia are the STOP-Bang, the P-SAP, the OSA50, and the DES-OSA scores [22–27]. These scores are illustrated in Fig 1. Prediction of hypoxemia is also clinically relevant. However, the STOP-Bang score does not predict hypoxemia in patients recovering from noncardiac surgery [28]. The ability of the three other scores has never been tested in this respect. We therefore compared the efficiency of these four scores to predict hypoxemia particularly in sOAS patients who are the patients at highest postoperative risk.

## Methods

**Fig 2:**
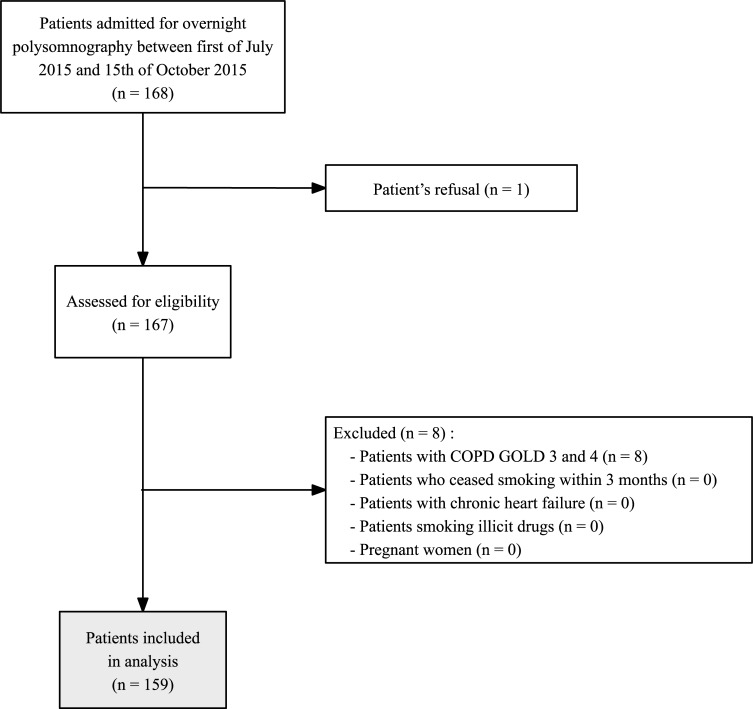
CONSORT trial profile.

This study was approved by the Ethics Committee of the Clinique Saint-Luc of Bouge (Clinique Saint-Luc of Bouge, Belgium, CE SLBO 2014/02, 20^th^ of April 2014; chairperson: Dr. van der Rest). Because the study was observational, the Ethics Committee requested oral informed consent only. We assessed 168 adult patients scheduled for an overnight PSG at the Clinique Saint-Luc of Bouge, Belgium, between the 1^st^ of July 2015 and the 15^th^ of October 2015, and finally included 159 patients (see Fig 2 for CONSORT trial profile). Exclusion criteria included: chronic obstructive pulmonary disease GOLD 3 and 4, insofar as those patients may experience diurnal hypoxemia independently from OSA [29], chronic heart failure, illicit drug intake or smoking (e.g. cannabis), and pregnancy. This study was registered with Clinical Trials (ref: NCT02050685).

Before PSG, all patients were weighed and measured to determine the body mass index (BMI). Parameters to calculate the four scores compared in this study were recorded by a blind investigator. Electrophysiological parameters and capillary oxygen saturation (SpO_2_) were monitored using the polysomnographic DREAM^®^ device (Medatec^®^, Brussels, Belgium). Signals were recorded on a dedicated computer using the Brainnet^®^ for Windows Software (Medatec^®^, Brussels, Belgium). The analysis of PSG data included the determination of sleep stages according to the *Rechtschaffen and Kales* scoring rules [30]. Periods of arousal were also considered, according to the American Sleep Disorders Association [31]. Criteria for the definition of apnea and hypopnea agreed with the American Academy of Sleep Medicine rules [32]. A hypopnea was defined as a reduction in airway flow ≥ 30% and associated with a SpO2 desaturation ≥ 4%. A second blind investigator collected the parameters derived from the PSG. As described in the Introduction, we only take into account severe OSA patients (patients with an AHI greater than 30 events per hour).

Hypoxemia is classically defined as a PaO_2_ less than 60 mmHg on room air [4]. SpO_2_ recording is usually performed during PSG. To the best of our knowledge, there is no unanimous definition of hypoxemia based on mean SpO_2_ during the night of a PSG. We therefore combined criteria used by others authors [7, 33, 34]. Hypoxemic patients were defined as those with a mean peripheral saturation in oxygen (SpO2) during sleep < 90% or with more than 10% of their sleep time with a SpO2 < 90%.

### Statistical analysis

The threshold values used for the present study were: a score ≥ 5 for the STOP-Bang score, a score ≥ 4 for the P-SAP score, a score ≥ 5 for the OSA50 score, and a score ≥ 7 for the DES-OSA score. The analyses of the PSG indicate that a positive test is the combination of severe OSA and nocturnal hypoxemia. The ability of the four scores to detect hypoxemic sOSA patients was compared using sensitivity (Se), specificity (Sp), Youden Index (YI) which was calculated as: [YI = sensitivity + specificity– 1] and varied in a range between -1 and 1, positive predictive value (PPV), negative predictive value (NPV), Positive Likelihood Ratio (+LR), Negative Likelihood Ratio (-LR), and area under ROC curve (AUROC) analyses. We also used the *Cohen Kappa* coefficient to assess the degree of concordance between clinical scores and the probability of hypoxemic sOSA[35]. Se, Sp, PPV, NPV, +LR, -LR are expressed in percentages. AUROC and Cohen Kapa coefficient are expressed in values (range from 0 to 1). All the results are expressed with their 95% Confidence Intervals. The *Mc Nemar* test was applied to compare the sensitivities and specificities. A *z*-test was used to compare AUROC [36]. A two-tailed P value < 0.05 was considered statistically significant. Data were analyzed using XLSTAT for Mac^®^ (version 2015.5.01, Addinsoft SARL^®^, Paris, France) and MedCalc^®^ (version 15.11.0 MedCalc Software bvba^®^, Ostend, Belgium). For our Se/Sp analyses, we used the threshold values defined by the authors of each score as being indicative of severe OSA: ≥ 5 for STOP-Bang, ≥ 4 for P-SAP, ≥ 5 for OSA50, and ≥ 7 for DES-OSA) [22–26]. We estimated that a sample size of 146 patients would provide a 90% power for detecting a 20% difference in Se or Sp between groups at a Bonferroni corrected alpha level of 0.008 (6 paired between-score comparisons). 159 patients were finally included in this study.

## Results

The patients’ characteristics are presented in Table 1. Complete results of Se, Sp, YI, PPV, NPV, +LR, -LR are provided in the S1 Appendix and summarized in Table 2. The *McNemar* tests revealed that the Se [95% CI] of STOP-Bang (0.8 [0.65–0.9]) at detecting hypoxemic patients was significantly lower as compared to P-SAP (0.97 [0.86–1]), and OSA50 (1 [0.89–1]). The *McNemar* tests showed that the Sp of DES-OSA (0.58 [0.49–0.66]) was significantly higher than the one of STOP-Bang (0.37 [0.29–0.46]), P-SAP (0.3 [0.23–0.39]), and OSA50 (0.09 [0.05–0.16]). The highest YI was obtained for DES-OSA (0.45 [0.38–0.53]). It was significantly greater than the YI of STOP-Bang (0.18 [0.12–0.34]), P-SAP (0.28 [0.21–0.35], and OSA50 (0.09 [0.05–0.14]). The best PPV and +LR were obtained for the DES-OSA score, while the best NPV and–LR were provided by the OSA50 score. S2 Appendix summarizes the results for mild (AHI between 5 and 14,9 events per hour) and moderate (AHI between 15 and 29.9 events per hour) OSA.

**Table 1:** Patients characteristics.

**N**	159
**Age (year)**	55.8 ± 14.0
**Gender: Male / Female (%)**	68 / 32
**BMI (kg/m**^**2**^**)**	31.79 ± 12.07
**sOSA patients (%)**	51.6%
**Hypoxemic sOSA patients (%)**	25.2%

Data are mean ± SD, or %. BMI = Body Mass Index, sOSA = severe OSA patients.

**Table 2:** Ability of the four scores to predict hypoxemic sOSA patients. Efficiency is expressed in terms of sensitivity (Se), specificity (Sp), Youden Index (YI), positive predictive value (PPV), negative predictive value (NPV), positive likelihood ratio (+LR), and negative likelihood ratio (-LR).

		Ability to detect hypoxemic sOSA patients		
		Value	95% CI	
**STOP-Bang**	Se	**0.800^2^^,^^3^**	0.649	0.897
	Sp	**0.370^1^^,^^3^**	0.288	0.459
	YI	**0.179**	0.119	0.239
	PPV	**0.299**	0.212	0.386
	NPV	**0.846**	0.748	0.944
	+LR	**1.269**	1.032	1.562
	-LR	**0.541**	0.279	1.049
**P-SAP**	Se	**0.975^2^**	0.857	1.000
	Sp	**0.303^4^^,^^6^**	0.227	0.391
	YI	**0.278**	0.208	0.348
	PPV	**0.320**	0.237	0.402
	NPV	**0.973**	0.921	1.000
	+LR	**1.398**	1.230	1.589
	-LR	**0.083**	0.012	0.583
**OSA50**	Se	**1.000^3^**	0.893	1.000
	Sp	**0.092^3^^,^^5^^,^^6^**	0.051	0.160
	YI	**0.092**	0.047	0.137
	PPV	**0.270**	0.199	0.342
	NPV	**1.000**	1.000	1.000
	+LR	**1.102**	1.040	1.167
	-LR	**0.000**		
**DES-OSA**	Se	**0.875**	0.733	0.949
	Sp	**0.580^1^^,^^4^^,^^5^**	0.490	0.665
	YI	**0.455^1^^,^^4^^,^^5^**	0.378	0.532
	PPV	**0.412**	0.307	0.516
	NPV	**0.932**	0.875	0.990
	+LR	**2.083**	1.636	2.651
	-LR	**0.216**	0.094	0.496

Results are fraction with 95% Confidence Interval (95% CI). The *Mc Nemar* test was applied to compare Se and Sp. Statistical significance was set at *P* = 0.0083 (0.05/6). Significant difference between the 4 scores (P < 0.0083, S1 Appendix)

“1” between STOP-Bang and DES-OSA

“2” between STOP-Bang and P-SAP

“3” between STOP-Bang and OSA50

“4” between DES-OSA and P-SAP

“5” between DES-OSA and OSA50

“6” between OSA50 and P-SAP.

The highest values for each analysis (Se, Sp, PPV, NPV, YI and +LR) and the lowest values for -LR are indicated by hatched cells.

**Fig 3:**
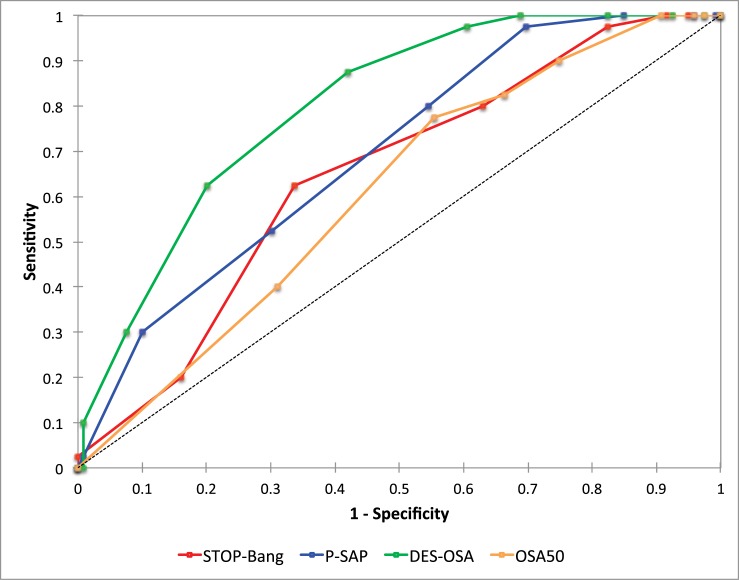
ROC curves for the four scores (STOP-Bang, P-SAP, OSA50, and DES-OSA) to predict hypoxemic sOSA patients.

Complete results of AUROC analyses are detailed in the S1 Appendix, summarized in Table 3, and illustrated in Fig 3. The AUROC of DES-OSA (0.8 [0.71–0.89]) was significantly larger than the AUROC of the three other scores, namely STOP-Bang (0.65 [0.54–0.75]), P-SAP (0.69 [0.59–0.79]), and OSA50 (0.61 [0.5–0.71]). Results of *Cohen Kappa* coefficient analyses are presented in Table 3. The highest *Kappa* value was obtained for DES-OSA (0.33 [0.21–0.45]) and was significantly different as compared to STOP-Bang (0.11 [0.01–0.21]), and OSA50 (0.05 [0.02–0.08]).

**Table 3:** Comparison of the areas under ROC curves (AUROC), as well as the Cohen Kappa Coefficients and their 95% Confidence Intervals of the four scores regarding for their ability to detect hypoxemic sOSA patients.

	STOP-BANG	P-SAP	OSA50	DES-OSA
AUROC (95% CI)	0.647 ^1^ (0.544 to 0.750)	0.695 ^2^ (0.595 to 0.795)	0.609 ^3^ (0.505 to 0.714)	0.801 ^1^^,^^2^^,^^3^ (0.713 to 0.888)
Cohen Kappa Coefficient (95% CI)	0.109 ^1^ (0.008 to 0.210)	0.165 ^4^ (0.092 to 0.238)	0.049 ^3^^,^^4^ (0.017 to 0.080)	0.331 ^1^^,^^3^ (0.211 to 0.451)

A *z-*test was applied to compare AUROC’s. The level of statistical significance was set at *P* = 0.0083 (0.05/6, S1 Appendix). 95% Confidence Intervals were used to compare Cohen Kappa Coefficients. Significant differences are indicated as follows

“1” between STOP-Bang and DES-OSA

“2” between DES-OSA and P-SAP

“3” between DES-OSA and OSA50

“4” between OSA50 and P-SAP.

## Discussion

Our findings suggest that the DES-OSA score is more efficient at identifying hypoxemic severe OSA patients than the STOP Bang, the OSA50, and the P-SAP scores. Whereas the STOP Bang, the OSA50, and the P-SAP scores were developed to detect all OSA patients (AHI ≥ 5) regardless of the potential risk of complications, the DES-OSA score more specifically identifies sOSA patients, who are at higher risk of postoperative complications, and for whom ambulatory surgery is questionable [2, 37, 38]. The better performance of DES-OSA in predicting the combination of sOSA and hypoxemia is, therefore, not surprising.

In our population, approximately half of the sOSA patients experienced nocturnal hypoxemia (sOSA = 51.2% of our population; combination sOSA and hypoxemia = 25.2% of our population). This confirms our previous findings [16]. Hypoxemic sOSA who cumulate risks to develop postoperative complications might represent an important subgroup of patients to identify preoperatively. However, so far, no score was assessed as to its ability to detect hypoxemic sOSA patients. The four clinical scores predictive of OSA compared in this study were selected because of their speed and ease of completion in a preoperative setting. The ideal score must be sensitive and specific enough to maximally identify hypoxemic sOSA patients, with a minimum of false positives. In this respect, the Youden Index, which corresponds to the sum of Se and Sp, is particularly of interest to compare the performance of the different scores. The DES-OSA score was associated with the highest Youden Index, significantly higher than those of the STOP-Bang, the P-SAP, and the OSA50 scores. The OSA50 score exhibits the lowest Youden Index which is significantly lower as compared to the DES-OSA and the P-SAP scores. Although our study did not concern preoperative patients, the clinical performance and relevance of the OSA50 score to detect hypoxemic sOSA patients during a preoperative screening is probably questionable. In a recent study, Khanna et al. reported that the STOP-Bang score does not predict postoperative hypoxemia in a surgical population [28]. Our study in patients addressed to a sleep clinic considers nocturnal hypoxemia outside any surgical setting. However, we confirm the poor efficiency of the STOP-Bang score at detecting hypoxemic sOSA patients.

Our study has some limitations. The study was performed in patients referred to a sleep center, which introduces a selection bias. Patients were explored for medical reasons, most often suspicion of OSA, but also other medical conditions such as insomnia or restless leg syndrome, and not necessarily in a perioperative setting. Consequently, we had a higher incidence of OSA in our population, as compared to a normal preoperative population. Most studies involving OSA screening had also the same limitation [39]. Future studies are therefore required in surgical patients to extend and confirm our results. In addition to nocturnal hypoxemia evidenced during PSG, those studies should investigate the incidence of postoperative hypoxemia, like in the study of Khana et al. [40], which may contribute to several postoperative complications.

## Conclusion

In conclusion, outside a perioperative setting, the DES-OSA score appears more efficient than the STOP-Bang, the P-SAP, and the OSA50 scores at detecting specifically hypoxemic sOSA patients. Identifying those patients is clinically relevant since they are probably at high risk of postoperative complications. This identification could prompt a more appropriate and intensive perioperative management using resources such as continuous positive airway pressure, postoperative monitoring in post-anesthesia care or intensive care unit, to improve outcome and safety of these patients, whether inpatients or outpatients. Future studies should extend our results in surgical patients and investigate the potential benefits of a tailored perioperative management for hypoxemic sOSA patients.

## Supporting information

S1 Appendix Complete results of sensitivity, specificity, Youden Index, positive predictive value, negative predictive value, positive likelihood ratio, negtaive likelihood ratio. (DOCX)

S2 Appendix Statistics for mild and moderate OSA. (DOCX)

S3 Appendix Patient’s data. (PDF)
