# Supplementary material for: Comparison of clinical scores in their ability to detect hypoxemic severe OSA patients
Source: PLoS One. 2018 May 7;13(5):e0196270. doi: 10.1371/journal.pone.0196270 (PMC5937788; doi:10.1371/journal.pone.0196270)
Supplement: S1 Appendix — (DOCX) [file pone.0196270.s001.docx]

**APPENDIX 1**

**Sensitivities (Se), Specificities (Sp), Predictive Positive Value (PPV),**

**Negative Predictive Value (NPV), Positive Likelihood Ratio (+LR), Negative Likelihood Ratio (-LR), and Youden Index (YI).**

**Comparisons of STOP-BANG, P-SAP, OSA50 and DES-OSA**

The scores were tested in their ability to detect hypoxemic sOSA patients. Hereafter, score by score, are presented their respective Se, Sp, PPV, NPV, +LR, -LR and YI. The scores were tested for all cutoff value. Cutoff values selected by the respective authors are presented in bold and hatched cells. The *Mc Nemar* test was applied to compare the sensitivities and specificities. A *z*-test was used to compare AUROC between them. For the *Mc Nemar* test and for the *z*-test, to correct for multiple comparisons and to avoid type I errors, the level of statistical significance was set at *P* = 0.0083 (0.05/6) (*).

***1. STOP-Bang score***

| STOP-Bang value | Se | 95% CI | | Sp | 95% CI | | PPV | NPV | +LR | -LR | YI |
| --- | --- | --- | --- | --- | --- | --- | --- | --- | --- | --- | --- |
| 1 | 1.000 | 0.893 | 1.000 | 0.000 | 0.000 | 0.039 | 0.252 |  | 1.000 |  | 1.000 |
| 2 | 1.000 | 0.893 | 1.000 | 0.050 | 0.021 | 0.109 | 0.261 | 1.000 | 1.053 | 0.000 | 1.050 |
| 3 | 1.000 | 0.893 | 1.000 | 0.084 | 0.045 | 0.150 | 0.268 | 1.000 | 1.092 | 0.000 | 1.084 |
| 4 | 0.975 | 0.857 | 1.000 | 0.176 | 0.118 | 0.256 | 0.285 | 0.955 | 1.184 | 0.142 | 1.151 |
| **5** | **0.800** | **0.649** | **0.897** | **0.370** | **0.288** | **0.459** | **0.299** | **0.846** | **1.269** | **0.541** | **1.170** |
| 6 | 0.625 | 0.470 | 0.758 | 0.664 | 0.575 | 0.742 | 0.385 | 0.840 | 1.859 | 0.565 | 1.289 |
| 7 | 0.200 | 0.103 | 0.351 | 0.840 | 0.763 | 0.896 | 0.296 | 0.758 | 1.253 | 0.952 | 1.040 |
| 8 | 0.025 | 0.000 | 0.143 | 1.000 | 0.961 | 1.000 | 1.000 | 0.753 | +Inf | 0.975 | 1.025 |

***2. P-SAP score***

| P-SAP value | Se | 95% CI | | Sp | 95% CI | | PPV | NPV | +LR | -LR | YI |
| --- | --- | --- | --- | --- | --- | --- | --- | --- | --- | --- | --- |
| 0 | 1.000 | 0.893 | 1.000 | 0.000 | 0.000 | 0.039 | 0.252 |  | 1.000 |  | 1.000 |
| 1 | 1.000 | 0.893 | 1.000 | 0.008 | 0.000 | 0.052 | 0.253 | 1.000 | 1.008 | 0.000 | 1.008 |
| 2 | 1.000 | 0.893 | 1.000 | 0.042 | 0.016 | 0.098 | 0.260 | 1.000 | 1.044 | 0.000 | 1.042 |
| 3 | 1.000 | 0.893 | 1.000 | 0.151 | 0.097 | 0.228 | 0.284 | 1.000 | 1.178 | 0.000 | 1.151 |
| **4** | **0.975** | **0.857** | **1.000** | **0.303** | **0.227** | **0.391** | **0.320** | **0.973** | **1.398** | **0.083** | **1.278** |
| 5 | 0.800 | 0.649 | 0.897 | 0.454 | 0.367 | 0.543 | 0.330 | 0.871 | 1.465 | 0.441 | 1.254 |
| 6 | 0.525 | 0.375 | 0.670 | 0.697 | 0.609 | 0.773 | 0.368 | 0.814 | 1.735 | 0.681 | 1.222 |
| 7 | 0.300 | 0.181 | 0.456 | 0.899 | 0.830 | 0.942 | 0.500 | 0.793 | 2.975 | 0.779 | 1.199 |
| 8 | 0.025 | 0.000 | 0.143 | 0.992 | 0.948 | 1.000 | 0.500 | 0.752 | 2.975 | 0.983 | 1.017 |

***3. OSA50 score***

| OSA50 value | Se | 95% CI | | Sp | 95% CI | | PPV | NPV | +LR | -LR | YI |
| --- | --- | --- | --- | --- | --- | --- | --- | --- | --- | --- | --- |
| 0 | 1.000 | 0.893 | 1.000 | 0.000 | 0.000 | 0.039 | 0.252 |  | 1.000 |  | 1.000 |
| 2 | 1.000 | 0.893 | 1.000 | 0.025 | 0.006 | 0.076 | 0.256 | 1.000 | 1.026 | 0.000 | 1.025 |
| 3 | 1.000 | 0.893 | 1.000 | 0.042 | 0.016 | 0.098 | 0.260 | 1.000 | 1.044 | 0.000 | 1.042 |
| **5** | **1.000** | **0.893** | **1.000** | **0.092** | **0.051** | **0.160** | **0.270** | **1.000** | **1.102** | **0.000** | **1.092** |
| 6 | 0.900 | 0.762 | 0.965 | 0.252 | 0.183 | 0.338 | 0.288 | 0.882 | 1.203 | 0.397 | 1.152 |
| 7 | 0.825 | 0.676 | 0.915 | 0.336 | 0.258 | 0.425 | 0.295 | 0.851 | 1.243 | 0.521 | 1.161 |
| 8 | 0.775 | 0.622 | 0.878 | 0.445 | 0.359 | 0.535 | 0.320 | 0.855 | 1.397 | 0.505 | 1.220 |
| 10 | 0.400 | 0.264 | 0.554 | 0.689 | 0.601 | 0.765 | 0.302 | 0.774 | 1.286 | 0.871 | 1.089 |

***4. DES-OSA score***

| DES-OSA value | Se | 95% CI | | Sp | 95% CI | | PPV | NPV | +LR | -LR | YI |
| --- | --- | --- | --- | --- | --- | --- | --- | --- | --- | --- | --- |
| 1 | 1.000 | 0.893 | 1.000 | 0.000 | 0.000 | 0.039 | 0.252 |  | 1.000 |  | 1.000 |
| 2 | 1.000 | 0.893 | 1.000 | 0.025 | 0.006 | 0.076 | 0.256 | 1.000 | 1.026 | 0.000 | 1.025 |
| 3 | 1.000 | 0.893 | 1.000 | 0.076 | 0.039 | 0.140 | 0.267 | 1.000 | 1.082 | 0.000 | 1.076 |
| 4 | 1.000 | 0.893 | 1.000 | 0.176 | 0.118 | 0.256 | 0.290 | 1.000 | 1.214 | 0.000 | 1.176 |
| 5 | 1.000 | 0.893 | 1.000 | 0.311 | 0.235 | 0.399 | 0.328 | 1.000 | 1.451 | 0.000 | 1.311 |
| 6 | 0.975 | 0.857 | 1.000 | 0.395 | 0.312 | 0.485 | 0.351 | 0.979 | 1.611 | 0.063 | 1.370 |
| **7** | **0.875** | **0.733** | **0.949** | **0.580** | **0.490** | **0.665** | **0.412** | **0.932** | **2.083** | **0.216** | **1.455** |
| 8 | 0.625 | 0.470 | 0.758 | 0.798 | 0.716 | 0.861 | 0.510 | 0.864 | 3.099 | 0.470 | 1.423 |
| 9 | 0.300 | 0.181 | 0.456 | 0.924 | 0.860 | 0.961 | 0.571 | 0.797 | 3.967 | 0.757 | 1.224 |
| 10 | 0.100 | 0.035 | 0.238 | 0.992 | 0.948 | 1.000 | 0.800 | 0.766 | 11.900 | 0.908 | 1.092 |
| 13 | 0.000 | 0.000 | 0.107 | 0.992 | 0.948 | 1.000 | 0.000 | 0.747 | 0.000 | 1.008 | 0.992 |

***5. McNemar test for comparisons of sensitivities***

|  | STOP-Bang | P-SAP | OSA50 | DES-OSA |
| --- | --- | --- | --- | --- |
| STOP-Bang |  | **0.008 *** | **0.005 *** | 0.366 |
| P-SAP |  |  | 0.317 | 0.102 |
| OSA50 |  |  |  | 0.025 |
| DES-OSA |  |  |  |  |

***6. McNemar test for comparisons of specificities***

|  | STOP-Bang | P-SAP | OSA50 | DES-OSA |
| --- | --- | --- | --- | --- |
| STOP-Bang |  | 0.102 | **< 0.001 *** | **< 0.001 *** |
| P-SAP |  |  | **< 0.001 *** | **< 0.001 *** |
| OSA50 |  |  |  | **< 0.001 *** |
| DES-OSA |  |  |  |  |

***7. Comparisons of AUROC***

| P-Values | STOP-Bang | P-SAP | DES-OSA | OSA50 |
| --- | --- | --- | --- | --- |
| STOP-Bang | 1 | 0.174 | **0.001 *** | 0.362 |
| P-SAP |  | 1 | **0.002 *** | 0.080 |
| DES-OSA |  |  | 1 | **< 0.001 *** |
| OSA50 |  |  |  | 1 |
