# Supplementary material for: Comparison of clinical scores in their ability to detect hypoxemic severe OSA patients
Source: PLoS One. 2018 May 7;13(5):e0196270. doi: 10.1371/journal.pone.0196270 (PMC5937788; doi:10.1371/journal.pone.0196270)
Supplement: S2 Appendix — (DOCX) [file pone.0196270.s002.docx]

**APPENDIX 2**

Ability of the four scores to predict AHI > 5, AHI > 15, AHI > 30, and AHI > 30 hypoxemic patients in terms of sensitivity (Se), specificity (Sp), Youden Index (YI), positive predictive value (PPV), negative predictive value (NPV), positive likelihood ratio (+LR), and negative likelihood ratio (-LR).

Results are given in fraction with 95% Confidence Interval (95% CI). Significant results of between 4 scores comparisons (based on 95% CI) are indicated as follows: “1” between STOP-Bang and DES-OSA, “2” between STOP-Bang and P-SAP, “3” between STOP-Bang and OSA50, “4” between DES-OSA and P-SAP, “5” between DES-OSA and OSA50, “6” between OSA50 and P-SAP.

| **Ability to detect AHI > 5**  **(mild, moderate, and severe OSA)** | | | **Ability to detect AHI > 30**  **(moderate and severe OSA)** | | | **Ability to detect AHI > 30**  **(severe OSA)** | | | **Ability to detect AHI > 30**  **(severe OSA)**  **AND**  **hypoxemic patients** | | |  |  |
| --- | --- | --- | --- | --- | --- | --- | --- | --- | --- | --- | --- | --- | --- |
|  | | Value | 95% CI | | Value | 95% CI | | Value | 95% CI | | Value | 95% CI | |
| **STOP-Bang**  (cutoff value: score > 5) | Se | **0.725^3^** | 0.644 | 0.792 | **0.781^3^** | 0.695 | 0.847 | **0.805^3^** | 0.705 | 0.877 | **0.8^2,3^** | 0.649 | 0.897 |
|  | Sp | **0.667** | 0.452 | 0.828 | **0.6^3^** | 0.454 | 0.729 | **0.468^3^** | 0.360 | 0.578 | **0.37^1,3^** | 0.288 | 0.459 |
|  | YI | **0.391^1,2^** | 0.315 | 0.467 | **0.381^3^** | 0.306 | 0.456 | **0.272^3^** | 0.203 | 0.341 | **0.179** | 0.119 | 0.239 |
|  | PPV | **0.935** | 0.897 | 0.973 | **0.832** | 0.774 | 0.89 | **0.617** | 0.541 | 0.693 | **0.299** | 0.212 | 0.386 |
|  | NPV | **0.269^3^** | 0.2 | 0.338 | **0.519^3^** | 0.441 | 0.597 | **0.692^3^** | 0.62 | 0.764 | **0.846** | 0.748 | 0.944 |
|  | +LR | **2.174^3^** | 1.926 | 2.422 | **1.952^1,3^** | 1.74 | 2.164 | **1.512^1,3^** | 1.375 | 1.649 | **1.269** | 1.032 | 1.562 |
|  | -LR | **0.413^3^** | 0.336 | 0.49 | **0.365^3^** | 0.29 | 0.44 | **0.417^2,3^** | 0.34 | 0.494 | **0.541** | 0.279 | 1.049 |
| **P-SAP**  (cutoff value: score > 4) | Se | **0.833^6^** | 0.761 | 0.887 | **0.868^6^** | 0.793 | 0.919 | **0.915^6^** | 0.831 | 0.96 | **0.975^2^** | 0.857 | 1 |
|  | Sp | **0.667** | 0.452 | 0.828 | **0.489** | 0.350 | 0.630 | **0.39^6^** | 0.289 | 0.502 | **0.303^4,6^** | 0.227 | 0.391 |
|  | YI | **0.5^4,6^** | 0.422 | 0.578 | **0.357^6^** | 0.283 | 0.431 | **0.304^6^** | 0.233 | 0.375 | **0.278** | 0.208 | 0.348 |
|  | PPV | **0.943** | 0.907 | 0.979 | **0.811** | 0.75 | 0.872 | **0.615** | 0.539 | 0.691 | **0.32** | 0.237 | 0.402 |
|  | NPV | **0.378** | 0.303 | 0.453 | **0.595^6^** | 0.519 | 0.671 | **0.811^6^** | 0.75 | 0.872 | **0.973** | 0.921 | 1 |
|  | +LR | **2.5^6^** | 2.199 | 2.801 | **1.699^4,6^** | 1.53 | 1.868 | **1.498^4,6^** | 1.364 | 1.632 | **1.398** | 1.230 | 1.589 |
|  | -LR | **0.25** | 0.183 | 0.317 | **0.269^4,6^** | 0.2 | 0.338 | **0.219^2,6^** | 0.155 | 0.283 | **0.083** | 0.012 | 0.583 |
| **OSA50**  (cutoff value: score > 5) | Se | **0.957^3,5,6^** | 0.906 | 0.982 | **0.982^3,5,6^** | 0.933 | 0.999 | **1^3,5,6^** | 0.945 | 1 | **1^3^** | 0.893 | 1 |
|  | Sp | **0.238^5^** | 0.104 | 0.456 | **0.2^3,5^** | 0.108 | 0.341 | **0.143^3,5,6^** | 0.081 | 0.24 | **0.092^3,5,6^** | 0.051 | 0.16 |
|  | YI | **0.195^2,5,6^** | 0.133 | 0.257 | **0.182^3,5,6^** | 0.122 | 0.242 | **0.143^3,5,6^** | 0.089 | 0.197 | **0.092** | 0.047 | 0.137 |
|  | PPV | **0.892** | 0.844 | 0.94 | **0.757** | 0.69 | 0.824 | **0.554^5^** | 0.477 | 0.631 | **0.270** | 0.199 | 0.342 |
|  | NPV | **0.455^3,5^** | 0.378 | 0.532 | **0.818^3,5,6^** | 0.758 | 0.878 | **1^3,5,6^** | 1 | 1 | **1** | 1 | 1 |
|  | +LR | **1.255^3,5,6^** | 1.167 | 1.343 | **1.228^3,5,6^** | 1.146 | 1.31 | **1.167^3,5,6^** | 1.098 | 1.236 | **1.102** | 1.040 | 1.167 |
|  | -LR | **0.183^3,5^** | 0.123 | 0.243 | **0.088^3,5,6^** | 0.044 | 0.132 | **0^3,5,6^** | 0 | 0 | **0** | 0 | 0 |
| **DES-OSA**  (cutoff value: score > 7) | Se | **0.58^5^** | 0.496 | 0.659 | **0.649^5^** | 0.558 | 0.730 | **0.78^5^** | 0.678 | 0.857 | **0.875** | 0.733 | 0.949 |
|  | Sp | **0.762^5^** | 0.544 | 0.896 | **0.756^5^** | 0.611 | 0.858 | **0.727^5^** | 0.618 | 0.814 | **0.58^1,4,5^** | 0.49 | 0.665 |
|  | YI | **0.342^1,4,5^** | 0.268 | 0.416 | **0.405^5^** | 0.329 | 0.481 | **0.508^5^** | 0.43 | 0.586 | **0.455^1,4,5^** | 0.378 | 0.532 |
|  | PPV | **0.941** | 0.904 | 0.978 | **0.871** | 0.819 | 0.923 | **0.753^5^** | 0.686 | 0.82 | **0.412** | 0.307 | 0.516 |
|  | NPV | **0.216^5^** | 0.152 | 0.28 | **0.459^5^** | 0.382 | 0.536 | **0.757^5^** | 0.69 | 0.824 | **0.932** | 0.875 | 0.99 |
|  | +LR | **2.435^5^** | 2.144 | 2.726 | **2.656^1,4,5^** | 2.33 | 2.982 | **2.862^1,4,5^** | 2.503 | 3.221 | **2.083** | 1.636 | 2.651 |
|  | -LR | **0.552^5^** | 0.475 | 0.629 | **0.464^4,5^** | 0.386 | 0.542 | **0.3021^5^** | 0.231 | 0.373 | **0.216** | 0.094 | 0.496 |
