# Supplementary material for: Comparison of clinical scores in their ability to detect hypoxemic severe OSA patients
Source: PLoS One. 2018 May 7;13(5):e0196270. doi: 10.1371/journal.pone.0196270 (PMC5937788; doi:10.1371/journal.pone.0196270)
Supplement: S3 Appendix — (PDF) [file pone.0196270.s003.pdf]

Kg	m	Tour cou cm	"M ou F"	Cig	DTM cm	Péri abdo	Ronflement	Fatigue diurr	Apn obs	HTA	Diabète	Mallampati	STOP-Bang	P-SAP	DES-OSA	OSA50	Age	BMI	Diff Ev-SO Bi	Sp % Bin
105	1.74	47	M	0	7	125	1	1	1	0	0	2	6	5	7	10	64	34.6809354	0	1
100	1.75	54	M	0	8	117	1	0	1	0	0	2	5	5	8	10	62	32.6530612	0	1
104	1.69	51	M	0	6	122	1	1	1	0	1	4	7	7	10	10	67	36.4132909	1	1
155	1.8	47	M	50	8	137	0	1	1	1	1	3	7	7	10	7	58	47.8395062	1	1
61	1.62	35	F	0	5	86	1	1	1	0	0	1	4	3	2	7	80	23.243408	0	1
107	1.68	52	M	0	9.5	124	1	1	1	0	1	4	7	7	9	10	70	37.9109977	1	1
112	1.75	48.5	M	0	7	117	1	1	1	0	0	2	7	5	8	10	75	36.5714286	0	1
100	1.73	44.5	M	0	7	109	1	0	1	0	0	1	5	5	5	10	79	33.4124094	0	1
84	1.81	40	M	0	7	97	1	1	1	0	0	4	5	4	6	7	55	25.640243	0	1
95	1.76	44	M	0	4.5	102.5	1	1	1	0	0	3	6	7	10	10	61	30.668905	0	1
53	1.53	41	F	0	5	115	0	1	0	0	1	3	3	5	6	5	89	22.6408646	0	1
80	1.76	38	M	10	6	100	1	0	1	0	0	1	4	3	4	7	57	25.8264463	0	1
102	1.795	47.5	M	6	8.5	123	1	1	0	1	0	3	6	7	8	8	59	31.65711	0	1
98	0.78	47.5	M	0	8.1	122	1	1	1	1	0	3	8	7	10	10	69	161.078238	0	1
111	1.92	42	M	0	5	122	1	1	1	0	0	1	5	6	5	8	49	30.1106771	0	0
97	1.8	44	M	10	8	110	1	1	1	0	0	2	6	4	7	10	59	29.9382716	0	0
85	1.83	44	M	0	6	118	1	0	0	1	0	3	5	6	8	8	68	25.3814685	0	1
85	1.7	45	M	0	7	109	1	1	1	1	0	3	7	6	8	10	74	29.4117647	0	0
108	1.6	41	F	0	7	119	0	1	0	1	0	4	5	5	8	5	54	42.1875	0	1
93	1.85	46	M	0	8	117	1	0	1	0	1	2	5	5	6	10	70	27.1731191	0	0
86	1.55	39	F	20	6	103	1	1	0	1	0	3	5	5	7	8	65	35.7960458	0	1
112	1.72	44	F	0	5	112	1	1	1	0	0	3	6	6	8	10	51	37.8583018	1	1
80	1.7	38	F	10	3	125	1	1	0	1	0	3	4	5	7	8	83	27.6816609	0	1
80	1.57	38	F	12	6	120	1	1	1	0	0	3	4	4	7	10	83	32.4556777	0	1
94	1.69	39	F	0	7	102	1	1	0	1	0	3	4	5	6	8	53	32.9120129	0	0
59	1.63	38	F	20	7.2	87	1	1	1	1	0	2	5	3	4	7	66	22.2063307	0	1
108	1.7	45	M	0	7	122	1	1	0	0	0	2	6	5	7	8	64	37.3702422	0	0
77	1.7	46	M	0	6	114	0	1	0	0	0	2	4	3	7	5	86	26.6435986	0	1
59	1.54	38	F	0	10	93	1	1	0	1	0	4	4	4	5	8	73	24.8777197	0	1
110	1.73	45	M	0	7	126	0	0	0	0	0	2	4	4	7	5	68	36.7536503	0	1
152	1.9	48	M	0	8	138	1	1	1	0	0	1	6	4	7	8	28	42.1052632	1	1
61	1.59	36	F	30	7	87	1	1	1	0	0	1	3	2	1	5	49	24.1287924	0	0
82	1.73	43	M	0	7	113	1	0	1	0	0	2	5	4	6	10	64	27.3981757	0	1
65	1.63	37	F	0	7	84	1	1	1	1	0	2	5	3	3	7	61	24.4646016	0	0
105	1.83	48	M	0	9.5	113	1	1	1	1	0	3	6	7	8	8	48	31.3535788	0	1
103	1.74	51.5	M	0	9.5	119	1	1	1	0	1	4	6	7	9	10	66	34.0203461	0	0
104	1.7	48	M	0	6	126	1	1	0	0	1	2	6	6	8	8	70	35.9861592	0	1
87	1.625	38	F	0	7.7	104	1	1	1	0	0	1	4	3	3	10	65	32.9467456	0	0
92	1.73	41	M	30	4.5	105.5	1	1	0	0	0	4	4	6	9	6	30	30.7394166	1	1
107	1.83	44.5	M	0	8	109	1	1	1	0	0	4	5	5	8	8	41	31.9507898	1	1
88	1.75	45	M	0	6	112	1	1	1	0	1	2	6	5	8	10	51	28.7346939	0	1
82	1.63	42	M	0	7.5	103	1	1	1	1	0	2	7	6	6	10	74	30.8630359	0	1
80	1.63	42	M	0	7	105	1	1	1	0	0	1	6	5	4	10	62	30.1102789	0	0
101	1.68	43	F	0	5	125	1	1	1	0	1	4	5	7	8	8	43	35.7851474	0	0
70	1.68	37	F	5	6	97	1	1	1	1	0	2	5	3	4	10	71	24.8015873	0	0
68	1.83	42	M	20	7	85	1	1	1	0	0	1	6	4	3	7	55	20.3051748	0	0
95	1.83	40	M	30	5.5	96	1	1	1	0	0	4	5	5	8	7	57	28.3675237	0	0
68	1.68	38	M	0	9	89	1	1	1	1	0	1	6	4	3	7	75	24.0929705	0	0
125	1.85	46	M	0	6	126	1	0	1	1	0	1	6	6	6	8	47	36.5230095	0	0
106	1.92	44.5	M	25	5	104	1	1	1	1	0	2	7	6	8	10	52	28.7543403	0	0
95	1.6	43	F	0	7	113	1	1	0	1	1	3	6	7	7	8	52	37.109375	0	0
82.5	1.71	42	M	30	8	103	1	0	0	0	0	4	3	4	7	6	38	28.2138094	0	1
140	1.7	47	F	20	7	132	1	1	1	1	1	3	6	7	9	8	49	48.4429066	0	1
90	1.86	44	M	0	8.5	110	1	1	0	1	0	3	5	5	7	6	41	26.0145682	0	1
98	1.72	47	M	0	6	117	1	1	1	0	0	3	6	6	9	10	72	33.1260141	0	1
114	1.8	44	M	0	7	121	1	1	0	0	0	2	5	5	7	6	48	35.1851852	0	0
55	1.73	30	F	0	6	77	0	1	0	0	0	1	1	0	2	0	30	18.3768252	0	0
80	1.63	37	F	0	6	106	1	1	1	1	0	1	5	4	3	10	69	30.1102789	0	1
122	1.82	47	M	0	7	121	1	1	1	0	0	1	7	5	5	10	59	36.8313006	0	0
72	1.78	44	M	0	6	97	1	0	1	0	0	1	5	4	5	7	77	22.7244035	0	0
102	1.67	44	M	0	8.5	122	1	1	0	1	0	3	7	7	8	8	69	36.5735595	0	1
65.5	1.85	43	M	0	7.8	101	1	0	1	0	0	3	4	5	7	5	45	19.138057	0	1
120	1.83	47	M	0	6	123	1	1	1	0	0	3	6	5	9	8	34	35.8326615	0	1
106	1.68	46	M	0	6	137	1	1	0	0	1	3	6	7	9	8	84	37.5566893	0	1
101	1.73	45	M	0	8	118.5	1	0	1	1	0	2	6	6	7	10	61	33.7465335	0	0

101	1.8	44	M	0	8	121	1	1	0	0	0	2	5	5	7	8	61	31.1728395	0	0
64	1.65	33	F	0	7	89	1	0	0	0	0	2	2	2	3	8	54	23.5078053	0	0
66	1.7	37	F	0	6	88	1	1	1	1	0	1	5	3	2	7	68	22.8373702	0	0
100	1.8	48	M	0	4.5	124	1	1	1	0	0	2	6	6	9	10	58	30.8641975	0	0
93	1.73	44	M	0	9	110	1	0	1	1	0	3	6	7	8	10	71	31.0735407	0	0
104	1.61	47	M	6	8	133	1	1	0	1	1	1	7	7	6	8	63	40.1219089	0	0
93	1.6	42	F	0	6	107	1	1	1	1	0	1	7	5	4	10	57	36.328125	0	0
110	1.58	39	F	0	6	121	1	1	1	0	0	1	4	2	6	8	37	44.0634514	0	0
110	1.89	47	M	0	7	124	1	1	1	0	0	3	6	6	8	10	56	30.7942107	1	1
84	1.71	44	M	17	8	100	1	1	1	0	0	2	5	4	7	5	45	28.7267877	0	0
109	1.65	42	F	10	8.2	133	1	0	0	1	0	1	4	5	4	6	50	40.0367309	0	0
107	1.83	39.5	M	0	6.5	108	1	1	1	0	0	2	4	3	6	8	38	31.9507898	0	0
100	1.78	41	M	0	9	109	1	1	0	0	0	2	5	5	6	8	51	31.5616715	0	0
95	1.75	48	M	0	8	106	1	1	1	1	1	3	7	8	8	10	62	31.0204082	0	1
100.5	1.76	45	M	0	7.5	118	1	0	1	1	0	3	6	7	8	10	57	32.4444731	0	1
102	1.82	40	M	0	9	104	1	1	1	1	0	1	5	5	4	8	47	30.7933824	0	0
70.5	1.63	39	F	0	6.5	105	1	1	1	1	0	1	5	3	2	10	68	26.5346833	0	0
141	1.93	50	M	0	7	131	1	1	1	0	1	1	7	6	6	10	53	37.8533652	0	0
98.5	1.805	47	M	0	7.5	120	1	0	1	1	0	3	6	7	8	10	68	30.23304	0	0
125.6	1.69	64	M	20	4.5	17	1	0	0	0	0	4	5	7	13	5	50	43.9760513	0	0
70	1.68	39	M	5	7.5	101	1	1	0	0	1	3	4	5	6	5	59	24.8015873	0	0
80	1.66	34.5	F	0	7.5	94	1	1	0	0	0	2	2	2	4	6	49	29.0317898	0	0
118	1.75	40	F	0	8	129	1	1	1	1	0	2	5	3	5	8	42	38.5306122	0	0
55	1.42	35	F	0	8	90	1	1	0	0	0	3	3	3	4	8	75	27.2763341	0	0
83	1.69	45	M	0	8	107	1	1	1	1	0	2	7	5	7	10	63	29.0606071	0	0
88	1.73	42	M	0	8.5	112	1	1	0	0	0	3	5	5	7	8	62	29.4029202	0	0
83	1.76	41	M	0	8	96	1	1	1	0	0	1	5	4	3	5	45	26.794938	0	0
132	1.7	47	M	0	8	131	1	1	1	1	0	2	7	6	9	8	48	45.6747405	0	1
125	1.78	50	M	17	8	135	1	1	0	0	0	2	6	5	9	8	54	39.4520894	1	1
77.5	1.67	40.5	M	0	6	103	1	1	0	0	0	2	5	4	6	8	93	27.7887339	0	0
89	1.77	37.5	M	0	8.5	101.5	1	1	1	1	0	4	5	4	7	5	42	28.4081841	0	0
110	1.78	44.5	M	0	7.5	112	1	1	0	1	0	1	5	6	5	6	44	34.7178387	0	0
94	1.65	45	F	0	8	100	1	1	1	1	1	2	6	6	6	10	52	34.5270891	0	1
94	1.71	42.5	M	11	8.2	110	1	1	1	1	0	2	7	6	7	10	50	32.1466434	0	0
96	1.72	44	M	0	8	120	1	1	1	0	0	2	6	5	7	10	61	32.449973	0	0
123	1.8	52	M	0	7	132	1	0	1	1	1	2	7	7	8	10	61	37.962963	0	0
132	1.54	41	F	0	6	128	1	1	0	1	0	3	6	6	9	8	82	55.6586271	0	0
76	1.69	41.5	M	0	7.5	98	1	1	1	1	0	3	7	6	6	7	60	26.6097125	0	0
84	1.71	44	M	13	7	114	1	1	1	1	0	2	7	5	7	10	58	28.7267877	0	0
97	1.8	44	M	13	8	100	1	1	1	0	0	2	5	3	7	5	34	29.9382716	0	0
79	1.66	43	M	0	8.2	102	1	0	0	0	0	2	4	4	7	5	57	28.6688924	0	0
97	1.73	41	M	0	7	122	1	1	1	1	0	2	7	6	6	10	53	32.4100371	0	0
105	1.72	47	M	0	7	130	1	1	1	0	1	3	7	7	8	10	59	35.4921579	0	0
107	1.58	47	F	0	8.5	117	1	1	0	1	0	3	5	6	9	6	49	42.8617209	0	0
90	1.63	37.5	F	0	8	103	1	1	1	1	0	4	5	5	6	10	64	33.8740638	0	0
74	1.57	43.5	F	0	4.5	111	1	1	1	1	0	4	6	7	9	10	59	30.0215019	0	0
105	1.63	41	F	30	8.5	115	1	1	1	1	0	3	6	6	7	8	49	39.5197411	0	0
97	1.86	40	M	0	8.2	109	1	1	0	0	0	2	3	3	6	6	47	28.0379235	0	0
108	1.785	46	M	0	8.9	115	1	1	0	1	0	2	5	5	7	6	34	33.8959113	0	0
89.5	1.82	41	M	0	7	108	1	1	1	0	0	2	6	4	5	10	53	27.0196836	0	0
90	1.78	39	M	0	7	96	1	1	1	0	0	3	4	3	7	5	28	28.4055044	0	0
80	1.91	40	M	0	7	87	1	1	0	0	0	1	3	2	3	3	24	21.9292234	0	0
89	1.75	45	M	0	8.1	111	1	0	1	1	1	2	6	6	7	10	65	29.0612245	0	0
57	1.58	34.5	F	0	4.5	84	0	1	0	1	0	3	3	4	6	2	69	22.8328793	0	0
91	1.74	38	M	0	4	105	1	1	0	1	0	1	4	6	6	6	47	30.0568107	0	0
85	1.74	42	M	0	9.1	99	1	1	1	0	0	1	6	4	4	7	51	28.0750429	0	0
100	1.85	46.5	M	0	6	105.5	1	1	1	1	0	4	7	6	9	10	51	29.2184076	0	0
93	1.87	44	M	0	7	107	1	0	1	1	0	1	5	5	4	8	49	26.5949841	0	0
85	1.86	39.5	M	2	5	93	1	1	1	0	0	2	4	3	6	5	26	24.5693144	0	0
86	1.72	46	M	0	6.5	119	1	0	1	1	0	3	6	6	8	10	55	29.0697674	0	0
98	1.79	47	M	0	8.1	118	1	0	0	0	0	4	4	6	8	8	52	30.5858119	0	0
80	1.76	42	M	0	8	97	1	1	0	0	0	1	4	4	3	3	48	25.8264463	0	0
81	1.72	41	M	0	6	101	1	1	1	1	0	1	7	5	4	7	57	27.3796647	0	0
109	1.68	44	F	0	9.5	126	1	0	0	0	0	3	4	5	7	8	67	38.6196145	0	0
108	1.83	47	M	0	7	127	1	1	1	0	0	2	5	4	7	8	28	32.2493953	0	0
108	1.83	44	M	10	8	110	1	0	0	1	0	3	4	6	8	6	24	32.2493953	0	0

62	1.6	37	F	0	5	94	0	0	0	0	0	2	1	2	4	5	65	24.21875	0	0
62	1.53	45	F	0	5	80	1	1	0	0	0	4	4	5	7	5	52	26.4855397	0	0
92	1.65	44	M	0	6	110	1	1	1	1	0	2	6	6	8	8	44	33.7924702	1	1
76	1.6	47	M	0	7.5	106	1	1	1	0	0	2	6	4	7	10	60	29.6875	0	0
65	1.68	36	M	0	7	87	1	1	1	0	0	1	5	3	2	7	57	23.0300454	0	0
79	1.87	43	M	0	8.1	93	1	1	0	0	0	3	5	5	7	5	65	22.5914381	0	0
82	1.65	43	M	0	9	108	0	1	0	0	0	1	4	4	5	5	54	30.1193756	0	0
106	1.79	51	M	0	8	115	1	1	1	1	1	3	7	8	9	10	71	33.0826129	0	0
80	1.68	37	F	0	7	93	0	1	0	0	0	2	1	1	4	3	46	28.3446712	0	0
105	1.84	43	M	0	8	112.5	1	0	0	0	1	2	4	6	7	8	53	31.0137051	0	0
80	1.7	43.5	M	0	4.5	101	1	0	0	0	0	4	4	6	9	5	74	27.6816609	0	0
75	1.7	36	F	0	6	100	1	0	1	0	0	1	3	2	2	10	56	25.9515571	0	0
103	1.7	45	M	15	5	122	1	1	1	1	1	2	7	7	8	8	35	35.6401384	0	0
82	1.71	45	M	0	8	110	1	0	0	1	0	2	5	5	7	8	66	28.0428166	0	0
73	1.8	36.5	M	0	6	83	1	1	1	0	0	1	4	2	3	5	20	22.5308642	0	0
81	1.7	35	F	0	7	92	1	1	1	0	0	2	3	1	4	8	38	28.0276817	0	0
78	1.8	39.5	M	0	7.5	96	1	0	0	0	0	3	2	3	6	3	27	24.0740741	0	0
80	1.67	40	F	0	4.5	89	0	1	0	0	0	4	1	2	8	3	42	28.6851447	0	0
46	1.55	30	F	0	7	76	0	1	0	1	0	1	3	2	1	2	61	19.1467222	0	0
72.5	1.88	38	M	0	7	77	1	1	0	0	0	2	3	2	5	3	32	20.5126754	0	0
63	1.6	37	F	0	7	79	0	1	0	0	0	1	1	1	1	0	45	24.609375	0	0
65	1.75	37	F	15	7.5	84.5	1	1	1	0	0	2	3	2	3	5	46	21.2244898	0	0
86.5	1.6	40	F	0	7.2	106	1	1	1	0	0	3	4	4	6	10	58	33.7890625	0	0
88	1.56	42	F	0	5.5	117	1	1	1	1	0	2	7	6	6	10	52	36.1604208	0	0
56	1.54	28	F	0	7	78	0	1	0	0	0	3	1	1	4	0	43	23.6127509	0	0
63.5	1.7	34.5	F	0	6.5	77	1	0	1	0	0	2	2	2	3	5	48	21.9723183	0	0
139	1.68	38	F	0	5.5	129	1	1	0	1	0	1	5	5	6	8	56	49.2488662	1	0
105	1.7	39	F	0	8	115	1	1	0	1	0	2	4	3	5	6	42	36.3321799	0	0
